# Other PHAC publications

**DOI:** 10.24095/hpcdp.46.6.05

**Published:** 2026-06

Other PHAC publications

https://doi.org/10.24095/hpcdp.46.6.05

Notice in the HPCDP Journal licensed under a Creative Commons Attribution 4.0 International License

**Researchers from the Public Health Agency of Canada also contribute to work published in other journals and books. Look for the following articles published in 2026:**

**Amaratunga K**. What remains. JAMA. 2026;335(7):578-9. https://doi.org/10.1001/jama.2025.23936

Andreacchi AT, Carnide N, Fuller A, **Blair A**, Siddiqi A, Shahidi FV. Socioeconomic inequities in drug poisoning deaths in Canada. Can J Public Health. 2026. https://doi.org/10.17269/s41997-026-01166-1

Biswas A, Chen C, **Lang JJ**, Villeneuve PJ, Smith PM, **Prince SA**. The interplay of home and work neighbourhood environment characteristics and associations with active commuting. J Transp Health. 2026;48:102278. https://doi.org/10.1016/j.jth.2026.102278

Marín-Jiménez N, Bizzozero-Peroni B, Molina-Garcia P, Ortega FB, Chaput JP, Zhang K, **Lang JJ**, et al. Clinical importance of simple muscular fitness tests to predict long-term health conditions: a systematic review and meta-analysis of 94 cohort studies. Br J Sports Med. 2026:bjsports-2024-109173. https://doi.org/10.1136/bjsports-2024-109173

